# IFNγ-dependent silencing of TFF1 during *Helicobacter pylori* infection

**DOI:** 10.1098/rsob.220278

**Published:** 2022-12-14

**Authors:** D. Eletto, F. Mentucci, M. Vllahu, A. Voli, A. Petrella, F. Boccellato, T. F. Meyer, A. Porta, A. Tosco

**Affiliations:** ^1^ Department of Pharmacy, University of Salerno, Fisciano, Salerno, Italy; ^2^ PhD Program in Drug Discovery and Development, University of Salerno, Fisciano, Salerno, Italy; ^3^ Ludwig Institute for Cancer Research, Nuffield Department of Clinical Medicine, University of Oxford, Oxford OX3 7DQ, UK; ^4^ Department of Molecular Biology, Max Planck Institute for Infection Biology, Berlin, Germany; ^5^ Laboratory of Infection Oncology, Institute of Clinical Molecular Biology, Christian Albrecht's University of Kiel—University Hospital Schleswig Holstein—Campus Kiel, Kiel, Germany

**Keywords:** helicobacter, trefoil factor 1, interferon-gamma

## Abstract

Chronic *Helicobacter pylori* infection is the leading cause of intestinal-type adenocarcinoma, as prolonged *Helicobacter* colonization triggers chronic active gastritis, which may evolve into adenocarcinoma of the intestinal type. In this environment, cytokines play a significant role in determining the evolution of the infection. In combination with other factors (genetic, environmental and nutritional), the pro-inflammatory response may trigger pro-oncogenic mechanisms that lead to the silencing of tumour-suppressor genes, such as trefoil factor 1 (TFF1). The latter is known to play a protective role by maintaining the gastric mucosa integrity and retaining *H. pylori* in the mucus layer, preventing the progression of infection and, consequently, the development of gastric cancer (GC). Since TFF1 expression is reduced during chronic *Helicobacter* infection with a loss of gastric mucosa protection, we investigated the molecular pathways involved in this reduction. Specifically, we evaluated the effect of some pro-inflammatory cytokines on TFF1 regulation in GC and primary gastric cells by RT-qPCR and luciferase reporter assay analyses and the repressor role of the transcription factor C/EBPβ, overexpressed in gastric-intestinal cancer. Our results show that, among several cytokines, IFNγ stimulates C/EBPβ expression, which acts as a negative regulator of TFF1 by binding its promoter at three different sites.

## Graphical abstract

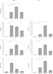

## 1. Introduction

In the first half of the twentieth century, stomach cancer was one of the leading causes of death in the USA and Europe [1]. Over the past decades, despite the reduction in the incidence of mortality due to gastric cancer (GC), this pathology remains a severe worldwide concern.

According to the Lauren classification, GC is classified into two major histological subtypes, diffuse-type and intestinal-type adenocarcinoma. Chronic *Helicobacter pylori* infection is the leading cause of intestinal-type adenocarcinoma, which accounts for 90% of non-cardia gastric carcinoma [2]. Even though *Helicobacter* infects up to 50% of the worldwide population, less than 1% of infected hosts develop gastric carcinoma, probably due to differences between bacterial strains, host genetics, non-genetic host features (e.g. age and gender) and environmental factors [3]. The initial event triggered by prolonged *Helicobacter* infection is chronic active gastritis which may evolve into adenocarcinoma of the intestinal type when the pathogen is not cleared properly by the host organism. The persistence of the bacterium within the gastric mucosa leads to an inflammatory response that, combined with oncogenic activation, promotes tumorigenesis.

The onset of the inflammatory processes involves the activation of the pattern recognition receptors (PRRs) of gastric epithelial cells by bacterial pathogen associated molecular patterns such as lipopolysaccharide, proteins, metabolites and nucleic acids [4]. The recognition of the foreign antigens by PRRs leads to intracellular signalling pathways triggering the release of proinflammatory cytokines. Subsequently, chronic inflammation is established, characterized by polarization of T helper Th1/Th17 cells, followed by the activation of CD4^+^CD25^+^ regulatory T (Treg) cells that control the inflammatory process [5].

The inflammatory response varies between groups of patients and seems to be strongly influenced by age [6]. In general, a Th1 response is predominant in adults, along with high expression of interferon γ (IFNγ), tumour necrosis factor α (TNFα), Interleukin-1 β (IL1β) and Interleukin-8 (IL8) [7–9], which are primarily responsible for the recruitment of neutrophils and foster setup of an inflammatory milieu. As well established, persistent inflammation promotes increased cellular turnover and the selection pressure of cells prone to malignant transformation [10].

In this context, cytokines play a significant role in determining the cell fate and the evolution of the infection. In particular, the balance between pro- and anti-inflammatory cytokines seems crucial in maintaining stomach mucosa homeostasis. This scenario may activate oncogenic pathways (NF-κB, AP-1, PI3K, STAT3, Wnt/β-catenin, COX2 etc.) [11] and silence tumour-suppressor genes (E-cadherin, RASSF1A, p16, GSTP1, SOCS1, SFRP1, PTEN, TFF1) [12].

Among these, TFF1 plays a crucial role and is considered a gastro-specific tumour suppressor since *Tff1^KO^* mice spontaneously develop adenomas in gastric antral/pyloric mucosa and about 30% progress to carcinoma [13]. Moreover, TFF1 is lost in about 40–60% of GCs for chromosome deletions, somatic mutations or promoter hyper-methylation [14]. *In vitro* studies of TFF1 expression upon *Helicobacter* infection always reveal an upregulation of the gastrointestinal protein [15,16]. Still, in a mouse model of infection, we recently demonstrated that TFF1 expression is upregulated during the acute phase of infection while gradually silenced when inflammation becomes chronic [16]. TFF1 belongs to the Trefoil factor family together with TFF2 and TFF3. All three are expressed in the mucous layer covering the gastrointestinal epithelium and contribute to its protective role. TFF1 is predominantly expressed in gastric foveolar cells and surface epithelial cells throughout the stomach, TFF2 within the gastric glands of the corpus and antral stomach regions, in gastric mucous neck cells and in the duodenal Brunner's gland, while TFF3 is predominantly expressed within goblet cells in the small intestine and colon [17].

Here, we analyse the molecular mechanisms involved in silencing TFF1, one of the protective and tumour-suppressing genes, in the context of the chronic inflammation triggered by *H. pylori* infection, focusing on the role of IFNγ, one of the major cytokines expressed during this inflammatory response.

## 2. Material and methods

### 2.1. Cell cultures

KATO III cells (gastric carcinoma, derived from a metastatic site and poorly differentiated) and THP-1 cells (human monocytic cell line derived from an acute monocytic leukemia patient) were obtained from the American Type Culture Collection (ATCC, Manassas, VA, USA).

Blood samples were collected from healthy unrelated volunteers (aged 25–40 years) at San Giovanni di Dio e Ruggi d'Aragona University Hospital, Salerno (Italy). Human peripheral blood mononuclear cells (PBMCs) were isolated by Ficoll-Paque PLUS gradient density (Cytiva, no. 17144002, Sweden).

All cells were maintained in RPMI 1640 (Euroclone, no. ECM2001L, Italy), supplemented, respectively, with 20% (v/v) fetal bovine serum (FBS, Euroclone, no. ECS0180D, South America, origin EU approved) for KATO III cells and 10% for the remaining cells, penicillin-streptomycin solution (100 U ml^−1^ penicillin and 100μg ml^−1^ streptomycin) (Euroclone, no. ECB3001D, Italy) and were grown at 37°C with 5% CO_2_ in a humidified incubator.

Primary cells for mucosoid cultivation were obtained from fresh gastric tissues resected from obese patients at the Helios Klinikum (Berlin-Buch, Germany). Tissues were obtained with the approval of the Ethics Committee of the Charité University Medicine, Berlin (EA1/129/12).

Gastric mucosoids, 2.5 × 10^5^ cells, were directly derived from freshly isolated glands or organoids, resuspended in 200 µl of culture medium prepared as described in [18], seeded into a collagen-coated (15 µg cm^−2^) (Gibco, no. A10644-01, Sweden) transwell inserts (Millipore, no. PIHP01250, Germany) placed into a 24-well plate. Mucosoids were grown at 37°C with 5% CO_2_ in a humidified incubator. After 3 days, the medium overlying the cells was removed from the well insert to start the air–liquid interface (ALI) culture, while the medium below the filter was replaced twice a week.

### 2.2. Bacterial cultures and infection experiments

*Helicobacter pylori* P12 strain was cultured as described previously [19]. Briefly, bacteria were grown on selective Columbia agar (Oxoid, Basingstoke, Hampshire, UK, no. CM0331) containing 7% (v/v) defibrinated horse blood (Thermo scientific, no. SR0050D, The Netherlands) supplemented with an antibiotic mix (Oxoid, no. SR0147E, UK). Bacteria plates were incubated for 3–4 days in a capnophilic atmosphere with 10% CO_2_. For infection experiments, bacteria were scraped from the plate using brain heart infusion (BHI Oxoid, no. CM1135, UK) with 10% FBS and measured at 600 nm (OD_600_), considering 1 OD_600_ = 1 × 10^8^ bacteria ml^−1^.

For conditioned medium experiments, PBMCs (1 × 10^6^ cells well^−1^) or THP-1 (2 × 10^6^ cells well^−1^), grown in 12-well plates, were infected with *H. pylori* at MOI 1 : 5. Cells and media were collected at different time points (24, 48, 72 h) for subsequent analyses.

Regarding primary cells, mucosoids were infected with *H. pylori* at MOI 1 : 50 and collected after 1, 3 and 5 days post-infection.

### 2.3. Cytokines' stimulation

KATO III cells were seeded into a six-well plate (6 × 10^5^ cells/well) for cytokine stimulation experiments, and the day after, stimulated, respectively, with 10 ng ml^−1^ IFNγ (Invitrogen, no. BMS303, Austria), 40 ng ml^−1^ TNFα (Invitrogen, no. BMS301, Austria) and 40 ng ml^−1^ IL1β (Invitrogen, no. RIL1BI, USA) for 24 h alone or in combination.

Mucosoids were cultured for two weeks, infected with *H. pylori* as described before, and then stimulated 24 h post-infection with 10 ng ml^−1^ IFNγ, 10 ng ml^−1^ TNFα and 5 ng ml^−1^ IL1β for 5 days.

### 2.4. RNA extraction and qRT-PCR

Total RNA was extracted using TRIzol reagent (Invitrogen, no. 15596018, New Zealand) following the manufacturer's instructions, and 1 µg of each RNA was retrotranscribed by M-MLV Reverse Transcriptase (GeneSpin S.r.l, no. STS-MRT, Italy). The real-time PCR was performed using the Light Cycler 480 II instrument (Roche, Basel, Switzerland). Suitable dilutions of cDNA were used for each gene in a 12 µl reaction using Luna Universal qPCR Master Mix (New Englands BioLabs, no. M3003, USA). The primers sequences are reported in electronic supplementary material, table S1. Results from three independent experiments in technical duplicates were analysed using the Delta-Delta CT method and HPRT1 as a reference gene.

### 2.5. Western blot analysis

For protein analysis, cells were resuspended with 1× gel-loading buffer (50 mM Tris–Cl pH 6.8; 2% w/v SDS; 0.1% bromophenol blue; 10% (v/v) glycerol and 100 mM β-mercaptoethanol), and sonicated (1 min, 10 s pulse on, 10 s pulse off, amplitude 30%, Vibra-Cell Sonics). Protein samples were then incubated at 100°C for 5 min, centrifuged at 10 000 g for 30 s, loaded onto a polyacrylamide gel for electrophoretic separation, and then transferred to Amersham Protran Premium 0.45 µm NC (GE Healthcare Life Sciences, no. GE10600003, Germany) nitrocellulose membrane. Ponceau red staining for 10 min was used to evaluate the transfer efficiency (0.1% solution in 1% v/v acetic acid). After blocking with 5% w/v of non-fat dry milk (BioRad) for 1 h, the membranes were incubated overnight with primary antibodies at 4°C. After washing three times with Tris-buffered saline with 0.05% Tween 20 for 10 min each, the membranes were incubated with secondary antibodies (Donkey anti-Rabbit (Jackson) and Donkey anti-Mouse (Jackson), respectively) for 1 h at room temperature, washed three times, and then visualized by LAS 4000 (GE Healthcare, Life Sciences) digital imaging system. The band densities were quantified by ImageJ (National Institutes of Health, USA). The antibodies are shown in electronic supplementary material, table S2.

### 2.6. Cell transfection

For the reporter-luciferase assays, KATO III cells (1.5 × 10^5^ cells /well) were seeded in a 24-well plate and, after 24 h, transfected by Lipofectamine 2000 reagent (Invitrogen, no. 11668-027, USA) following the manufacturer's instruction. Different pGL3 plasmids (Promega, no. E1751, USA) (pGL3-1kb-Luc, pGL3-0.8kb-Luc, pGL3-0.5kb-Luc, pGL3-0.3kb-Luc and pGL3-0.2kb-Luc) containing several deletions of the TFF1 promoter sequence (from −931 bp, −830 bp, −583 bp, −306 bp and −212 bp) upstream of a Luciferase reporter gene, were used for these experiments. Cells were co-transfected with 1 µg of each plasmid and 0.1 µg of a plasmid containing the β-galactosidase gene, whose expression was used as a normalization parameter of transfection efficiency.

For Small-interfering RNA knockdown experiments, KATO III cells were seeded into a 12-well plate at a density of 1 × 10^5^ cells well^−1^ and transfected with C/EBP-β siRNA (60 nM) (Santa Cruz Biotechnology, no. sc-29229, USA) or control siRNA (60 nM) (Santa Cruz Biotechnology, no. sc-37007, USA) by Lipofectamine 2000.

### 2.7. Dual-luciferase reporter assay

The luciferase activity of transfected cells was measured using the Luciferase/β-Galactosidase Luciferase Assay Kit, Dual-Light (Applied Biosystems). The relative transcriptional activity was calculated as Firefly Luc/β-galactosidase. The analysis was performed in quadruplicate, and the results are reported as the ratio of firefly luciferase activity and β-galactosidase (relative transcriptional activity). The light emission was measured by EnSpire Alpha Multimode Plate Reader (PerkinElmer).

### 2.8. Chromatin immunoprecipitation

Chromatin immunoprecipitation (ChIP) assays were performed using a SimpleChIP Enzymatic Chromatin IP Kit (Magnetic Beads) (Cell Signaling, no. 9003, USA), according to the manufacturer's protocol. Briefly, KATO III cells were treated with 1% paraformaldehyde for protein-DNA crosslinking and then neutralized with glycine (Cell Signaling, no. 7005S, USA). Samples were treated for nuclei extraction and DNA shearing with Micrococcal Nuclease (Cell Signaling, no. 10011, USA) for 20 min at 37°C to obtain DNA fragments of approximately 150–900 bp. Nuclei were sonicated to break the nuclear membrane, chromatin was immunoprecipitated with anti-C/EBPβ antibody (Cell Signaling, no. 90081S, USA), or negative control without antibody overnight at 4°C, and then incubated for 2 h with ChIP-Grade Protein G Magnetic Beads (Cell Signaling, no. 9006, USA). DNA was eluted, de-crosslinked for 2 h at 65°C, and purified. The binding of C/EBPβ to the TFF1 promoter was assessed by PCR using the primers reported in electronic supplementary material, table S1. The results were analysed using per cent input method. Briefly, the Ct value was adjusted for the input sample (2% of total DNA used) and expressed as a percentage of the total input chromatin.

### 2.9. Enzyme-linked immunosorbent assay

PBMCs isolated from healthy donors (1 × 10^6^ cells per well) were cultured in a final volume of 1 ml of medium without antibiotics in 12-well plates and infected with *H. pylori* (MOI 1 : 5) for different time points (24 h, 48 h, 72 h). Cells were collected and centrifuged at 300 g for 5 min. The pellets were washed with PBS (Euroclone, no. ECB4004L, Italy) and used for RNA or protein extraction. Supernatants were collected for the quantification of the following cytokines by ELISA according to the manufacturer's instructions: IFNγ (Invitrogen, no. 88-7316-88, USA), IL6 (Diaclone SAS, no. 950.030.192, France), TNFα (Diaclone SAS, no. 950.090.192, France) and IL1β (Diaclone SAS, no. 850.006.192, France).

### 2.10. Immunofluorescence analysis

Mucosoids either in Wnt (+W) and RSPO (+R) conditions or undifferentiated (−W, −R) were fixed overnight in 4% paraformaldehyde at 4°C and filters washed and embedded orthogonally in Histogel (HG-4000-144) using casting mould. Then, the paraffin blocks were generated inside a casting mould on a Paraffin console (Microm), and 5 µm sections were cut with a paraffin rotation microtome (Microm).

Sample slides were processed as described in [18] and probed with anti-TFF1 (LSBIO, no. LS- LS-C155659). The slides were analysed by confocal microscopy using a Leica TCS SP-8 microscope. Images were analysed with FIJI software.

### 2.11. Statistical analysis

The results are expressed as means ± s.d. Data were statistically analysed using the unpaired Student *t*-test. Statistical analyses and graphs were carried out using PRISM4 software (GraphPad Software, La Jolla, CA, USA). A *p*-value < 0.05 was considered statistically significant.

## 3. Results and discussion

### 3.1. Role of the immune system in regulating TFF1 expression

TFF1 is a well-known protective factor of the gastric mucosa, whose expression is modulated by *Helicobacter* infection; in particular, *in vivo* experiments in a mouse model showed that during *Helicobacter* infection, the peptide was first induced and then gradually reduced when the inflammation becomes chronic [16]. On the other hand, all *in vitro* systems revealed an upregulation of TFF1 upon *Helicobacter* infection despite its duration. These observations suggest a potential role of the host immune response in the TFF1 silencing during the late phase of infection.

**Figure 1.**
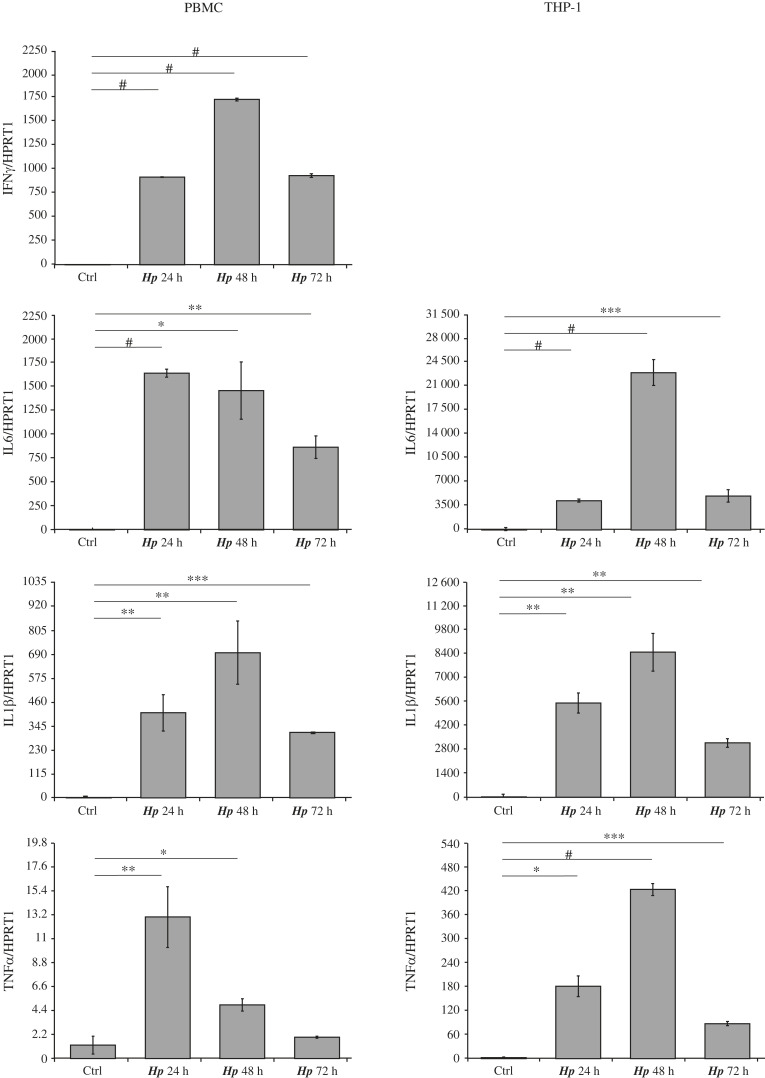
Cytokines expression in PBMC or THP-1 cells infected by *H. pylori*. PBMC or THP-1 cells were infected with *H. pylori* at MOI 1 : 5 and cells were collected at 24-, 48- and 72 h post-infection. The indicated cytokines were measured by real-time PCR analysis. HPRT1 was used as a housekeeping gene. Graphs are representative of at least three independent experiments. Data are expressed as mean ± s.d. (*t*-test, *p-value ≤ 0.05, **p-value ≤ 0.01, ***p-value ≤ 0.001, ^#^*p*-value ≤ 0.0001), and the mean of controls was set to 1.

**Figure 2.**
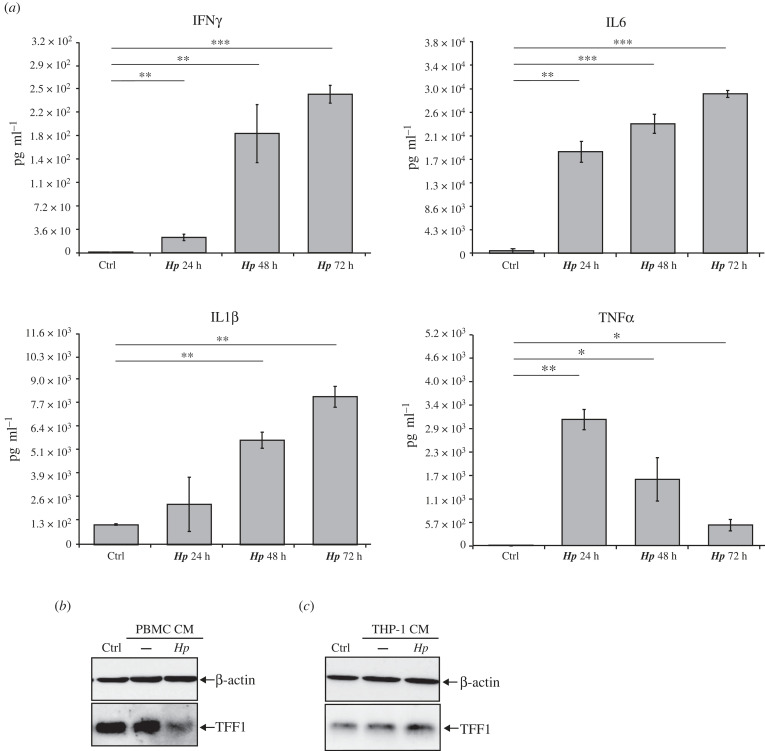
TFF1 expression is affected by conditioned media from *H. pylori*-infected immune cells (*a*) ELISA analysis of the indicated cytokines from PBMCs conditioned media. Data are expressed as mean ± s.d. (*t*-test, * *p*-value ≤ 0.05, ** *p*-value ≤ 0.01, *** *p*-value ≤ 0.001). (*b*,*c*) Western blot analysis of intracellular TFF1 protein in KATO III cells incubated with conditioned media (CM), respectively, from PBMCs (*b*) or THP-1 (*c*) cells infected with *H. pylori* at a MOI 1 : 5 for 72 h.

Following this hypothesis, we analysed TFF1 expression in gastric KATO III cells incubated with different conditioned media of immune cells infected with *H. pylori*. In particular, we used THP-1 cells (a human monocyte leukaemia cell line) and PBMCs (human peripheral blood mononuclear cells). Both models were infected with *H. pylori* at a MOI 1 : 5, and cells and media were collected after different time points. The most significant cytokines, reported to be highly expressed during *Helicobacter* infection, were measured by real-time PCR (figure 1) and ELISA assay (figure 2*a*). The analysis of cytokines' content either by ELISA or RTqPCR revealed a significant difference between the two immune models. Figures 1 and 2*a* show that the *H. pylori* infection activates PBMCs and THP-1, expressing high levels of each analysed cytokine (IL8, IL6, TNFα and IL1β) except for IFNγ, undetectable in THP-1 cells. Indeed, the ELISA analysis did not reveal IFNγ protein in THP-1 media at any time of the infection, whereas the same cytokine was highly expressed by PBMCs (figure 2*a*).

Since the higher accumulation of cytokines in the media was observed in samples collected at 72 h post-infection, KATO III cells were incubated with 72 h-conditioned media respectively from each immune cell culture, and TFF1 expression was measured 24 h post-incubation. According to the results in figure 2*b,c*, TFF1 was differently regulated depending on the immune cells. In particular, it was dramatically downregulated in KATO III cells incubated with media from *Hp*-infected PBMCs, while conditioned media from THP-1 did not affect its expression.

### 3.2. Cytokines are involved in TFF1 silencing during *H. pylori* infection *in vitro*

**Figure 3.**
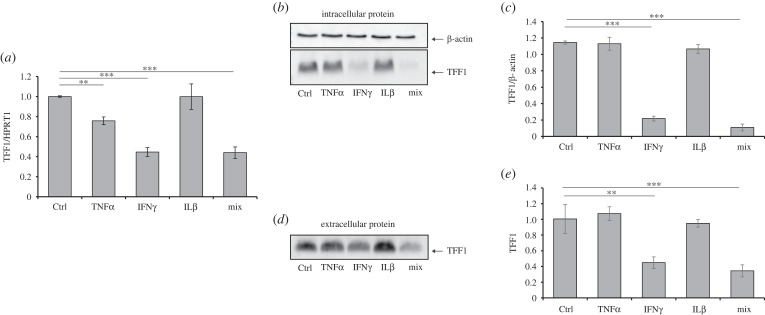
TFF1 expression is downregulated by IFNγ. (*a*) Real-time PCR analysis of TFF1 in KATO III cells after 24 h incubation with TNFα (40 ng ml^−1^), IFNγ (10 ng ml^−1^) and IL1β (40 ng ml^−1^) alone or in combination (mix). HPRT1 was used as a housekeeping gene. (*b*,*c*) Western blot analysis of intracellular TFF1 protein in KATO III cell line upon the above-described treatments and densitometric analysis of TFF1 signals (*b*) normalized to β-actin. (*d*,*e*) Western blot analysis of secreted TFF1 protein from the supernatants of KATO III treated as (*a*) and the densitometric analysis of TFF1. Images are representative of at least three independent experiments. Data are expressed as mean ± s.d. (*t*-test, **p-value ≤ 0.01, ***p-value ≤ 0.001), and the mean of the controls was set to 1.

To further confirm that the TFF1 downregulation promoted by *H. pylori*-infected PBMCs conditioned media was explicitly due to IFNγ, KATO III cells were directly incubated with some of the most induced cytokines during *Helicobacter* infection [20], TNFα, IL1β and IFNγ, alone or in combination for 24 h, and TFF1 expression was analysed both at the mRNA and at the protein level. The most relevant reduction was observed with IFNγ or the combination of the three cytokines. As shown in figure 3*a*, TFF1 mRNA was significantly reduced by TNFα and IFNγ, alone or in combination with the other selected cytokines. Moreover, when we analysed the intracellular (figure 3*b,c*) and the secreted protein (figure 3*d,e*), we observed a significant reduction only upon incubation with IFNγ, either in the single or the triple treatment. Moreover, the effect was maintained over time as downregulation of TFF1 persisted up to 7 days of IFNγ incubation (electronic supplementary material, figure S1).

This evidence confirmed that TFF1 downregulation following co-incubation with PBMCs conditioned media was mainly due to the IFNγ.

**Figure 4.**
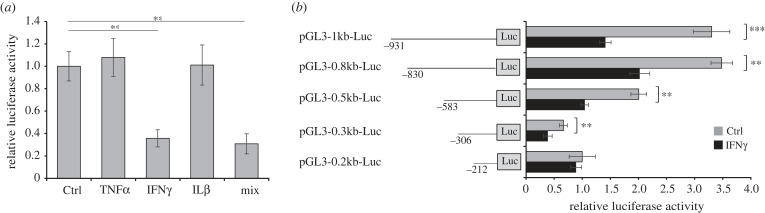
IFNγ reduces luciferase activity under TFF1 promoter control. (*a*) Luciferase reporter assays in KATO III cells transfected with pGL3-1kb-Luc plasmids, containing a fragment starting from −931 bp of TFF1 promoter upstream of a luciferase reporter gene and incubated with TNFα (40 ng ml^−1^), IFNγ (10 ng ml^−1^) and IL1β (40 ng ml^−1^) alone or in combination (mix). (*b*) Luciferase reporter assays in KATO III cells transfected with different plasmids containing fragments of various lengths from −931 bp to −212 bp and stimulated with IFNγ for 24 h. Data are expressed as the mean of four independent experiments ± s.d. (*t*-test, **p-value ≤ 0.01, ***p-value ≤ 0.001), the mean of pGL3-0.2kb-Luc plasmid was set to 1.

To explore whether the TFF1 downregulation by IFNγ was due to a direct effect on its promoter, we performed a luciferase reporter assay using a plasmid containing the TFF1 promoter region from −1036 bp up to −17 bp. To this aim, we transfected KATO III cells with the pGL3-1kb-Luc plasmid containing the TFF1 promoter upstream of the luciferase reporter gene and incubated them with TNFα, IL1β and IFNγ, alone or in combination for 24 h. As shown in figure 4*a*, the reporter activity was significantly inhibited by IFNγ, alone or in combination with the other cytokines. Once assessed that the TFF1 promoter was sensitive to IFNγ, we aimed to identify the minimal repressor region by measuring the luciferase activity of different fragments of the TFF1 promoter under IFNγ stimulation. In figure 4*b*, the activity of each promoter fragment was significantly reduced by IFNγ to the same extent except for the fragment containing the region from −212 bp to −17 bp, suggesting that elements sensitive to IFNγ are present in the region between −931 bp and −212 bp of TFF1 promoter.

### 3.3. TFF1 downregulation depends on C/EBPβ

**Figure 5.**
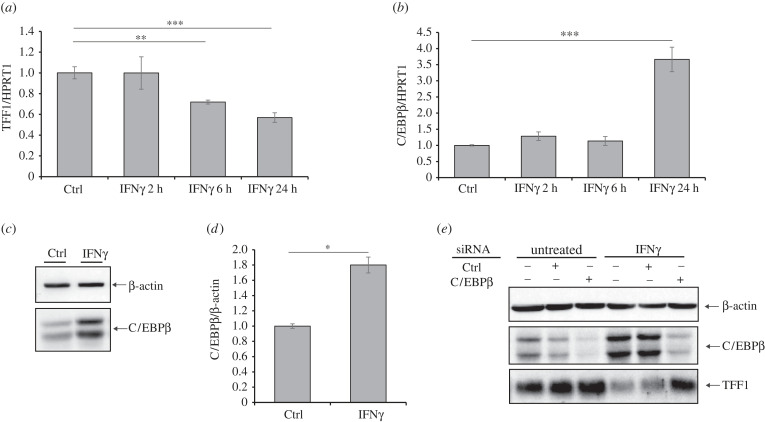
IFNγ induces C/EBPβ expression, which in turn represses TFF1. (*a*,*b*) KATO III cells were stimulated with 10 ng ml^−1^ IFNγ, and at the indicated time points, TFF1 and C/EBPβ transcripts were measured by RTqPCR. HPRT1 was used as a housekeeping gene. Data are expressed as the mean of three independent experiments ± s.d. (*t*-test, **p-value ≤ 0.01, ***p-value ≤ 0.001). (*c*) C/EBPβ protein level was measured by western blot analysis in KATO III cells incubated with 10 ng ml^−1^ IFNγ for 24 h. (*d*) Densitometric analysis of C/EBPβ from (*c*) normalized to β-actin. Images are representative of three independent experiments. Data are expressed as mean ± s.d. (*t*-test, * *p*-value ≤ 0.05). (*e*) KATO III cells were transfected with small interfering RNA (siRNA) control (Ctrl) or C/EBPβ and exposed to 1 ng ml^−1^ IFNγ for 24 h. Protein expression of C/EBPβ and TFF1 was revealed by western blot analysis. β-actin served as a loading control.

IFNs mediate the immune responses in vertebrates to viral or microbial infections by inducing the transcription of several IFN-stimulated genes (ISGs). The two kinds of IFNs, type I (IFNα/β) and type II (IFNγ), bind to distinct cell-surface receptors and promote signals that stimulate the expression of ISGs. In particular, the binding of IFNγ to its receptor recruits the Janus kinases JAK1 and JAK2, which enable the phosphorylation of STAT1 [21]. STAT1 dimers rapidly migrate to the nucleus and induce the expression of ISGs. One of the ISGs is the transcription factor CCAAT/enhancer-binding protein-β (C/EBPβ), a bZIP factor that can bind as a homo- or hetero-dimer to specific DNA regions, regulating several functions, such as normal tissue development, cellular function, cellular proliferation and differentiation [22]. This factor is also implied to play a pro-oncogenic role in several types of cancer, where it seems to promote cell proliferation and impair apoptosis. Since it is frequently overexpressed in intestinal-type GC, it is hypothesized that its activation might represent an upstream event of tumorigenesis. Its function has been investigated in murine stomachs, where it would favour carcinogenesis by repressing TFF1 expression [23]. Following this evidence, we verified whether TFF1 down regulation by IFNγ occurred via C/EBPβ. We first measured the C/EBPβ expression during IFNγ stimulation at the transcriptional and protein levels in gastric KATO III cells (figure 5*a–d*). We found an inverse correlation between TFF1 and C/EBPβ transcript levels during IFNγ time-course incubation (figure 5*a*,*b*). The C/EBPβ protein was twofold upregulated by IFNγ, confirming that this factor is an ISG also in this cellular system. More importantly, to assess the involvement of C/EBPβ in the regulation of TFF1 expression upon IFNγ stimulation, we silenced C/EBPβ by small interfering RNA and found that IFNγ-depending downregulation of TFF1 was prevented (figure 5*e*). Altogether, these data suggest that stimulation with IFNγ leads to up regulation of C/EBPβ which in turn represses TFF1 expression.

### 3.4. C/EBPβ represses TFF1 transcription by binding its promoter

**Figure 6.**
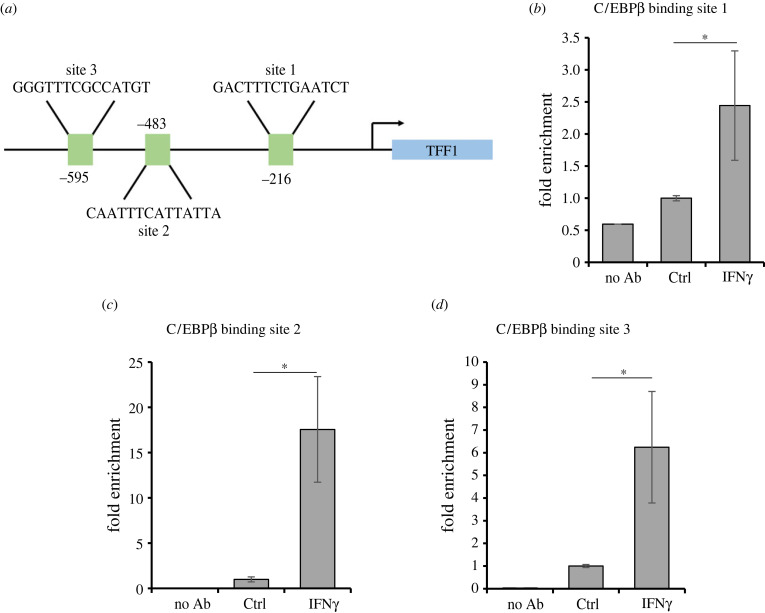
C/EBPβ binds to TFF1 upon IFNγ stimulation. (*a*) Schematic of C/EBPβ binding sites on TFF1 promoter. Site 1 (−216 bp) was identified by Sankpal *et al*. [24], while sites 2 (−483 bp) and 3 (−585 bp) are considered putative binding elements. (*b*–*d*) KATO III cells were stimulated with 10 ng ml^−1^ IFNγ for 24 h, and the binding of C/EBPβ to TFF1 promoter was analysed by ChIP followed by RTqPCR analysis. Data are expressed as the mean of three independent experiments ± s.d. (*t*-test, *p-value ≤ 0.05).

C/EBPβ is a known transcription factor that regulates multiple cell functions by binding DNA upon stimulation. We asked whether IFNγ favours its translocation other than inducing its expression. As shown in electronic supplementary material, figure S2, most of the protein is already in the nucleus, at steady state conditions, and the stimulation with IFNγ seems only to increase its level in this cellular compartment. We therefore asked whether IFNγ promotes its binding to the TFF1 promoter. To this aim, we performed a ChIP assay with an anti-C/EBPβ antibody and evaluated the binding to the TFF1 promoter by RTqPCR on three potential binding sites (figure 5*a*). The first one, at −216 bp (named here site 1), has been identified as a C/EBPβ binding site by Sankpal [24], and the remaining two are putative sites, retrieved by TRANSFAC [25], here named site 2 (−483 bp) and 3 (−585 bp). Stimulation with IFNγ significantly enhances the C/EBPβ binding specifically to all three sites on the TFF1 promoter, even if to different extents (figure 6*b–d*), without any effect on another region here used as a negative control (electronic supplementary material, figure S3).

### 3.5. Pro-inflammatory cytokines reduce TFF1 expression in primary cells

**Figure 7.**
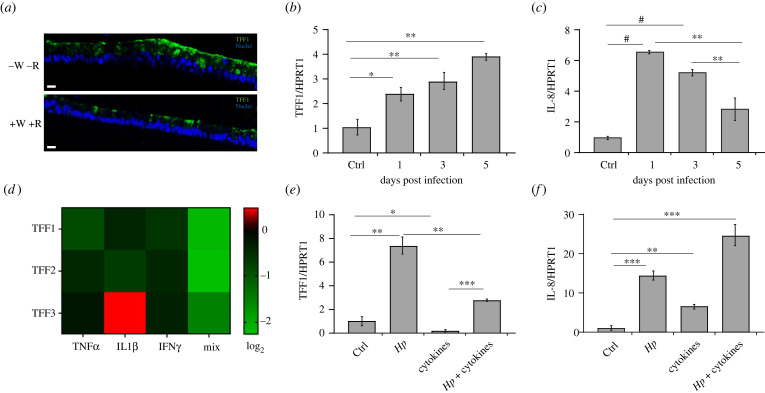
TFF1 expression in mucosoids is affected by cytokines during *Helicobacter* infection. (*a*) Immunofluorescence analysis of TFF1 (green) in ‘foveola’ (without Wnt3A/RSPO1, −W−R, *a*–*c*) and in ‘basal' cells (with Wnt3A/RSPO1, +W +R, *d­*–*f*) of mucosoids. Scale bars: 10 µm. (*b*) TFF1 and (*c*) IL8 expression level in mucosoids after 1, 3 and 5 days post *H. pylori* infection. HPRT1 was used as a housekeeping gene. Data are expressed as the mean of three independent experiments ± SD (*t*-test, **p-value ≤ 0.01, ****p-value ≤ 0.0001). (*d*) Microarray analysis of TFF1, TFF2 and TFF3 in mucosoids in +W +R conditions exposed to the indicated cytokines for 5 days. (*e*,*f*) Mucosoids were infected with *H. pylori* at MOI 50 and, the day after, exposed to the mixture of cytokines (TNFα, IL1β and IFNγ) for 5 days. (*e*) TFF1 and (*f*) IL8 transcripts were measured by RTqPCR. HPRT1 served as a housekeeping gene. Data are expressed as the mean of three independent experiments ± s.d. (*t*-test, **p-value ≤ 0.01, ***p-value ≤ 0.001).

To overcome the multiple limitations of tumoural cell systems and confirm the results in primary cells, we enrolled mucosoids as a model of *Helicobacter* infection [18]. They present multiple advantages compared to organoid cells or two-dimensional (2D) monolayer cultures, as they are long-lived, polarized epithelial monolayers, and more importantly, they support long-term infection with *H. pylori*. Moreover, by modulating Wnt signalling or coculturing with a defined stromal population, mucosoid cells can be differentiated into foveolar or basal phenotypes. Interestingly response of basal versus foveolar cells to *H. pylori* infection is distinct as cells at the gland base show a much more robust inflammatory response compared to foveolar cells. Since our data show an inverse correlation between TFF1 expression and inflammatory response during *Helicobacter* infection [16], we asked whether the different inflammatory response between basal and foveolar cells was also due to a different expression of TFF1 in these two compartments. For this aim, mucosoids were cultured for 6 days either in the presence of recombinant Wnt3A (+W) and R-spondin (RSPO)1 (+R) or deprived of both Wnt3A/RSPO1 (−W−R) to differentiate them respectively into basal or foveolar cells. Immunofluorescence analysis revealed that TFF1 is preferentially expressed in foveolar cells (−W−R) (figure 7*a*), confirming that this primary cellular system also shows an inverse correlation between TFF1 expression and inflammatory response following *H. pylori* infection.

Following the previous evidence, to better define the role of TFF1 in gastric mucosoids during *H. pylori* infection, we monitored how the gastric factor is modulated in the basal glands infected for 5 days. As shown in figure 7*b*, the TFF1 transcript is significantly upregulated over time during the infection. Interestingly, the induction of IL8 peaked at 24 h and then gradually reduced over time as TFF1 increased (figure 7*c*), confirming that the host organism responds to infection by expressing protective factors, such as TFF1, that would limit the inflammatory response triggered by *H. pylori*.

As performed in KATO III, mucosoids were stimulated with IL1β, TNFα and IFNγ, alone or in combination (triple treatment) for 5 days and subjected to microarray analysis (figure 7*d*). The heatmap shows an extract of the data of TFF1, TFF2 and TFF3 from Traulsen *et al*. (manuscript in preparation). Both TNFα and IFNγ reduce TFF1 expression, and the triple treatment lowered its expression even more. Moreover, the other two trefoil factors, particularly TFF2, were also profoundly downregulated by the triple treatment. Indeed, Dossinger and co-workers [25] showed a direct effect of C/EBPβ on the promoters of all three factors. The effect of the single cytokines differed on the three factors suggesting that the immune system uniquely regulates each gene.

Finally, to recapitulate the phenotype observed during chronic *Helicobacter* infection *in vivo*, which causes TFF1 downregulation, mucosoids were first infected with *H. pylori* and, the following day, exposed to the cytokines mentioned above (TNFα, IL1β and IFNγ) mimicking the activation of the host immune system (figure 7*e,f*). As expected, the infection stimulates TFF1 expression, as observed previously, and conversely, the stimulation with the cytokines represses its expression limiting the over-expression promoted by *H. pylori* (figure 7*e*). As a hallmark of the infection, IL8 transcript was measured in the same samples and found stimulated by the infection, and its expression increased even more by the co-treatment with the cytokines (figure 7*f*), confirming that a low expression of TFF1 corresponds to a higher inflammatory response.

## 4. Discussion

The integrity of the gastric mucosa is preserved by a subtle balance between protective and aggressive factors. The first line of defense is represented by mucus-buffer phospholipid layers that maintain a neutral pH at the surface epithelial luminal interface; whereas the second line of defense is constituted by the surface epithelial cells, which produce mucins, prostaglandins, trefoil peptides, peptide growth factor, and their receptors, heat shock proteins and β-defensins. Belonging to the trefoil factor family is TFF1, which is involved in mucosal protection, together with mucins, by inducing repair and helping the formation of a stable mucus gel layer [26]. It is also known for binding to *Helicobacter*, which may favour the tropism of the bacteria within the human gastric mucosa [27]. More recently, TFF1 has been reported to induce the formation of aggregates in which bacteria lose their spiral shape and exhibit reduced motility, suggesting that TFF1 might exert its protective role by slowing down the motion of *Helicobacter* towards the epithelium [28].

Another role of TFF1 is related to copper, which binds to the factor favouring the homodimer formation, considered the protein's active form [29]. Copper itself influences TFF1 expression, which, in turn, plays a role in gastric copper cell homeostasis [30]. We recently demonstrated that *Helicobacter* takes advantage of gastric copper, reducing its availability for the host, and worsening the infection outcome. Moreover, in copper-deprivation conditions, bacteria hardly colonize the epithelium, and inflammation is reduced, and this correlates with higher TFF1 levels [31], confirming its protective role during *Helicobacter* infection.

One of the molecular mechanisms revealing the anti-inflammatory activity of TFF1 during *H. pylori* infection has been recently reported by Soutto *et al*., who showed a reduced NFkB-dependent STAT3 activation promoted by TFF1 [32].

In *Helicobacter*-induced GC, the subjects undergo a corpus-predominant inflammation that develops into atrophic gastritis, intestinal metaplasia, and eventually intestinal-type adenocarcinoma along the Correa pathway [33]. We decided to analyse the molecular events that occur during chronic gastritis and predispose to the appearance of malignant lesions, such as the silencing of TFF1, which is one of the gastric-tumour suppressors.

Since we were not able to detect other than induction of TFF1 expression in cellular models of *Helicobacter* infection, even when using a primary culture of mucosoids, while, on the contrary, TFF1 resulted in being downregulated during chronic inflammation of mice stomachs infected by *Helicobacter felis*, we decided to explore the role of the immune system during the infection. To this aim, we used two different conditioned media from THP1 and PBMCs infected by *Helicobacter pylori,* and we observed a reduction of TFF1 expression only with PBMCs conditioned medium. The analysis of the most prominent pro-inflammatory cytokines involved in *Helicobacter* infection (IL8, IL6, TNFα, IL1β and IFNγ) revealed that the main difference between the two conditioned media was on IFNγ, not expressed in the THP1 system. We conclude that this monocytic cell line derived from an acute leukaemia is not a good model to mimic immune response upon *Helicobacter* infection.

To verify if IFN*γ* was directly involved in TFF1 silencing, we used the recombinant cytokine on KATO III, a tumour cell line that expresses high levels of the gastrointestinal protein, and confirmed that the treatment with the pro-inflammatory cytokine reduces both mRNA and protein levels of TFF1.

IFN*γ* is induced by the highly virulent strains of *H. pylori* [34] and plays an important role in the progression of *H. pylori*-driven epithelial pathology [35]. Accordingly, mice that lack IFN*γ* do not develop gland hyperplasia or metaplasia in response to infection; this pro-inflammatory cytokine produced by CD4^+^ CD25^−^ Th cells is crucial for the control of *Helicobacter* infection on the one hand and induces preneoplastic changes in the gastric mucosa on the other hand [36].

IFN*γ* not only directly affects epithelial cell fate but also interferes with stromal stem cell niche signalling by downregulating the expression of bone morphogenetic protein (BMP) signalling molecules, leading to upregulation of Rspo3 in the stroma and interfering with gland homeostasis [37].

In this context, *H. pylori* tries to block IFN*γ* signalling and escape the host inflammatory response reducing cholesterol levels in gastric epithelial cells [38]. When this mechanism fails, IFN*γ* binds to its receptors JAK1 and JAK2, inducing their dimerization and the activation of different phosphorylation cascades, which end with the transcriptional activation of several genes. Among them, the activation of the transcription factor C/EBPβ was described [39].

Given that C/EBPβ is frequently overexpressed in GC and is associated with the suppression of TFF1 [23], C/EBPβ activation may represent an upstream event with implications for gastric tumorigenesis, whose TFF1 downregulation can be a hallmark.

Our data demonstrate that there are three functional C/EBPβ binding sites on the TFF1 promoter and that IFN*γ* stimulation increases the levels of this transcription factor bound to all three elements, even if to different extents. Confirming the role of C/EBPβ, IFNγ-depending downregulation of TFF1 was prevented when this factor was silenced.

The use of the mucosoid cellular system revealed that TFF1 is more expressed in foveolar than in basal glands and correlates with a reduced inflammatory response measured in these cells upon *Helicobacter* infection [18]. In addition, we confirmed that TFF1 is also upregulated in the primary cellular system even after 5 days of infection, and its stimulation was limited in the presence of cytokines (TNFα, IL1β and IFNγ), confirming a crucial role of pro-inflammatory cytokines in the regulation of the gastric protective factor again.

Altogether, our results suggest that the silencing of one of the gastric tumour suppressors during *Helicobacter pylori*-induced chronic inflammation is promoted by the production of pro-inflammatory cytokines released by recruited activated immune cells. Among them, IFNγ seems to have an essential role in the regulation of TFF1 expression activating C/EBPβ, which binds the TFF1 promoter and represses its expression.

Nevertheless, the silencing of TFF1 may also be triggered by epigenetic modification, as DNA methylation and histone modifications, including H3K9 methylation and H3 deacetylation at the TFF1 promoter, as observed in a mouse model of carcinogenesis [40]. This aspect will be deepened in future experiments by taking advantage of the primary mucosoid cell system to dissect the time course of the arrival of different modifiers on the TFF1 promoter.

## Data Availability

The data are provided in the electronic supplementary material [41].
